# A Retrospective Audit of Osteoporosis Management Following Fragility Fractures in a Hospital Setting

**DOI:** 10.7759/cureus.76047

**Published:** 2024-12-19

**Authors:** Sushmit Singh, Shireen Patil, Myat Thein, Jigisha Kulkarni, Azra Z Kakroo, Ladlee Singh, Bhagyashree Dhotare, Elizabeth Nolan

**Affiliations:** 1 Orthopaedics, Warrington and Halton Teaching Hospitals NHS Foundation Trust, Warrington, GBR; 2 Medicine, Warrington and Halton Teaching Hospitals NHS Foundation Trust, Warrington, GBR; 3 Geriatrics, Warrington and Halton Teaching Hospitals NHS Foundation Trust, Warrington, GBR; 4 Medicine, University Hospitals Sussex NHS Foundation Trust, Worthing, GBR; 5 Anaesthesia, Wrightington, Wigan and Leigh Teaching Hospitals NHS Foundation Trust, Wigan, GBR; 6 Paediatric Surgery, Institute of Child Health, Kolkata, IND; 7 Internal Medicine, Warrington and Halton Teaching Hospitals NHS Foundation Trust, Warrington, GBR

**Keywords:** bisphosphonates, fragility fractures, geriatric care, osteoporosis, secondary prevention

## Abstract

Background

Fragility fractures, often caused by osteoporosis, are a major public health concern among the growing population of the United Kingdom (UK). In addition to being a major source of illness and mortality, the rising incidence of osteoporosis places a heavy strain on healthcare systems if it is not adequately managed. In order to lower the risk of additional fractures, current guidelines place a strong emphasis on the timely evaluation and treatment of fragility fractures.

Methods

This retrospective audit was carried out in Warrington Hospital, UK, to examine the management of fragility fractures in patients aged 65 years and above across two cycles (January to March 2022 and April to May 2023, respectively). All data was obtained from electronic medical records, and management was assessed, including treatment with bisphosphonates, vitamin D, dual-energy X-ray absorptiometry (DEXA) scans and further referrals for metabolic bone clinics.

Results

The first cycle included 144 inpatients and showed significant under-utilization with only 16% receiving bisphosphonates treatment (23 inpatient prescriptions), low referrals for DEXA scan and metabolic bone clinics. Assessment and supplementation of vitamin D was also suboptimal. The second cycle of the audit reviewed 165 patients and showed minor improvement in bisphosphonates prescription, although post-discharge rates were low with around 80% of patients (104 of 131, as 34 patients were prescribed while inpatient) not receiving medications. The DEXA scans and metabolic bone clinic referrals also remained inadequate in the second cycle.

Conclusions

This audit highlighted the significant gap in the management of fragility fractures and persistent deficiencies in spite of robust guidelines and interventions set by various bodies. The implementation of treatment guidelines for osteoporotic patients at hospital level can help in timely initiation of antiresorptive therapy, DEXA scans and utilisation of metabolic bone clinics to optimise outcomes and reduce the burden on healthcare systems. There is a need for further research and education to improve this adherence to set guidelines and improve outcomes.

## Introduction

Fragility fractures are defined by the fact that they can occur as a result of minor trauma, such as falls from a standing height or during ordinary activities, such as getting out of a chair or even something as simple as sneezing or coughing [1-4]. There are serious public health concerns since these fractures frequently signify the existence of underlying osteoporosis. Fragility fractures are especially harmful to people 65 years and above [5], and as the number of older adults rises, fragility fractures are expected to increase as well over the next several decades. The United Kingdom (UK) population is aging, and around 3.5 million people are estimated to have osteoporosis [6]; this predicted increasing prevalence is placing an increased financial strain on healthcare services.

Many studies have demonstrated that people who have a fragility fracture are much more likely to suffer another fracture, especially within the two years after the first injury, a condition known as imminent fracture risk [7,8]. This increased risk of further osteoporotic fractures highlights how crucial it is to start preventive treatment right from admission to hospital. In addition to significantly lowering the risk of subsequent fractures, early management also lessens the morbidity that may result from additional injuries [9]. Current clinical guidelines strongly advise that osteoporotic patients who have experienced a fragility fracture at any relevant site during the last two years begin medication immediately in order to address this urgent condition [10].

Organisations like the National Institute of Health and Care Excellence (NICE) have issued recommendations for the management of patients with fragility fractures that emphasise the importance of incorporating vitamin D level evaluation and supplementation and appropriate bone protection measures [11]. General practitioners are directly involved in diagnosis and treatment of such at-risk groups and work in tandem with fracture liaison services for overall management [12].

Hospitalisation of at-risk individuals provides a good chance for intervention even though community care and primary physicians are crucial for continuous management [13]. Since elderly patients are at high-risk category for osteoporosis and related fractures, clinicians should do further evaluation and provide prophylactic treatment during their hospital stay. The hospital environment provides thorough evaluation of bone health, thereby facilitating greater chance of assessment and treatment to decrease their risk of further fractures.

## Materials and methods

A retrospective audit was conducted at Warrington Hospital, a district general hospital in Warrington, UK, from January to May 2023 in two cycles. Patients with fragility fractures who were 65 years of age or older were the study's primary focus. The study excluded patients with atypical fractures, incomplete data, or insufficient follow-up. The electronic medical records of patients who were hospitalised for fragility fractures were used to retrieve data. As the audit data was quantitative, it was analysed using anonymised spreadsheet in Excel. This was used to ascertain the number and percentage of inpatients with non-adherence to national guidelines.

All of the patients' records, including clinic visits, surgical notes and discharge summaries, were carefully examined. Relevant data points were the type of fracture and mechanism of injury, results from dual-energy X-ray absorptiometry (DEXA) scans and the medications prescribed for osteoporosis, which comprised hormone therapies, antiresorptive medications, vitamin D and parathyroid analogues. Furthermore, vitamin D levels were examined to test the sufficiency of supplementation, and the Fracture Risk Assessment Tool (FRAX) score was computed for risk assessment [14]. The audit assessed the prescription of inpatient bisphosphonates, continuity of anti-resorptive treatment prescribed by general practitioners (GPs), requests for DEXA scans and referrals to bone metabolic clinics when indicated. Since, this patient group is more likely to have multiple comorbidities, a thorough study was done by at least two auditors to highlight any comorbidities preventing antiresorptive management and see if such patients were referred to metabolic bone clinic.

The Six Standards of Hip Care, which recommend that all patients who present with a fragility fracture be assessed for the necessity of antiresorptive medication to avoid future fractures, were among the established standards that were used to evaluate the data collected [15]. Additionally, the National Osteoporosis Guideline Group's (NOGG) UK 2021 recommendations were used as a standard [16]. According to these guidelines, postmenopausal women and men 50 years of age and older who are at high or very high risk should be offered medication treatment for fracture prevention. The online NOGG intervention thresholds based on FRAX scores could direct treatment decisions in situations when bone mineral density (BMD) assessment is not practical, particularly for elderly patients with a history of fragility fractures.

We sought to ascertain the percentage of patients receiving vitamin D and antiresorptive therapy in compliance with the guidelines during the first audit cycle. Following patient discharge, we evaluated the gaps in antiresorptive therapy continuity in the community and found that secondary preventive techniques require major enhancements. Patients indicated for DEXA scans or consultations with bone metabolic clinics were also documented.

Following the initial audit cycle, the following interventions were carried out to improve adherence to guidelines. We worked towards enhancing education on osteoporosis management and standardising prescribing practices by forming local protocols in a simplified flowchart manner. We pushed for initiation of antiresorptive treatment prior to patient discharge by forming new protocols, and facilitating referrals for alternative treatment options before discharge. An older people osteoporosis clinic was set after the first cycle to help with bone clinic burden and patients to receive specialist orthogeriatrics input after fragility fractures. A re-audit was conducted in the second cycle, collecting retrospective data from April 12 to May 25, 2023, using the same inclusion and exclusion criteria, to measure the impact of the implemented recommendations after the first audit cycle.

## Results

The first cycle of the audit included 144 patients who were admitted from January to March 2022. The majority of these patients were suffering from neck of femur fractures (NOF), with 100 females and 44 males and average age of 82 years (range from 65 to 101). In adherence to the guidelines, we found FRAX score recorded for 54% (78 out of 144) of inpatients, 89 out of 144 patients (62%) had their vitamin D assessments and around 54% (78 out of 144) of patients were started on vitamin D replacement therapies.

In the first cycle, among the 144 patients, the most prevalent comorbidities were hypertension (32 patients) and diabetes (38 patients). Gastrointestinal conditions (oesophagitis, gastritis, ulcerative colitis, Crohn’s disease, hiatus hernia, Barret’s oesophagus, ulcers, diverticulitis, reflux disorders) were seen in 21 patients, and 15 patients presented with dementia and other neurologic diseases (Parkinson’s disease, multiple sclerosis, polyneuropathy). Other notable comorbidities included chronic obstructive pulmonary disease (COPD) in 10 patients, cardiac conditions in 23 patients and rheumatoid conditions in 16 patients, with eight receiving corticosteroid treatment. 

In terms of medications with potential fracture risks, 42 patients were on proton pump inhibitors (PPIs), and aromatase inhibitors were noted in three patients. Notably, no current use of hormone replacement therapy (HRT) was reported; however, two patients had previously discontinued it without being commenced on alternative osteoporosis therapies. Androgen blockers were used by two patients, and 16 were identified as smokers, a known risk factor for bone health.

Out of 144 patients, antiresorptive treatment in the form of bisphosphonates was initiated in only 23 patients (16%) during their hospital stay. Further study showed that following discharge, around 87% patients (105 out of 121, as 23 patients were started whilst inpatient) did not receive bisphosphonate treatment from their general practitioners. Majority of patients did not receive DEXA scans (110 out of 144) and only 25% (34 out of 144) of them were referred to metabolic bone clinic. The booking of DEXA scans and referrals to bone clinic were delayed due to reliance on GP referrals during the first audit cycle.

The first audit cycle was followed by the implementation of a series of interventions to enhance patient management. Regular clinical teaching sessions, multidisciplinary meetings and the development of simple, understandable protocols that were displayed in flowcharts were all examples of educational initiatives. These programs, which focused on following national guidelines, were directed at doctors, orthopaedic surgeons and allied health professionals. The main goals were to make sure that antiresorptive medication was started, perform DEXA scans and quickly refer patients to osteoporosis bone clinics.

To improve collaboration between geriatricians and orthopaedic surgeons, an Ortho-geriatrics Senior House Officer role was created in the trust ensuring accountability, communication and uniformity in patient care. The geriatrics team conducted regular multidisciplinary meetings and bedside teachings specifically designed for the frailty population to further support consistency. These meetings made sure that new rotation physicians were trained specifically to increase awareness. Additionally involved were the emergency physicians and the orthopaedic team.

**Figure 1:**
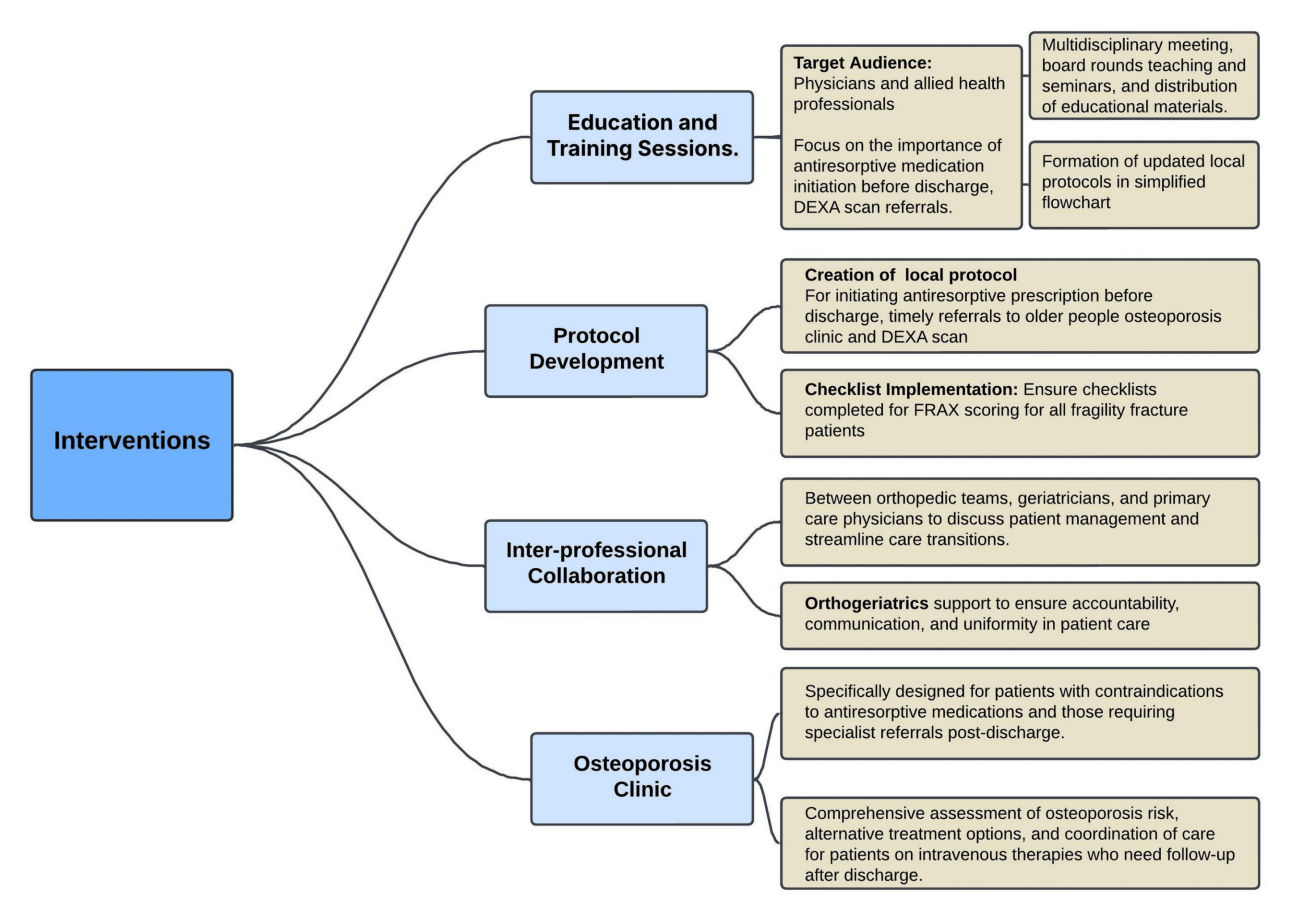
Interventions implemented after first cycle of the audit DEXA: dual-energy X-ray absorptiometry; FRAX: Fracture Risk Assessment Tool.

The immense caseload and long referral waitlist for metabolic bone clinics was leading to further delay in treatment. As a solution to this problem, an older people osteoporosis clinic was created. The goal of this clinic was to streamline investigations and treatment for patients with fragility fractures, facilitate specialist referrals and coordinate secondary prevention initiatives (Figure 1).

**Figure 2:**
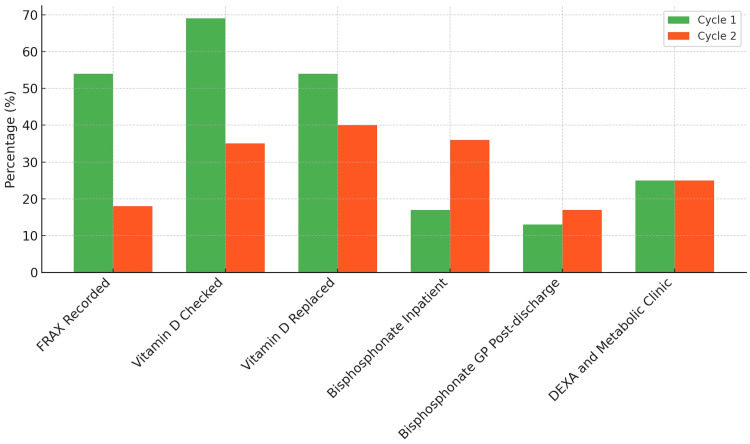
Comparison of data from the two audit cycles There was a minor improvement in inpatient bisphosphonates prescription in second audit cycle. FRAX: Fracture Risk Assessment Tool; GP: general practitioner; DEXA: dual-energy X-ray absorptiometry.

The second audit cycle collected data from April to May 2023, encompassing 165 patients with an average age of 77 years (range from 65 to 89). There were 127 females and 38 male patients in this cycle. Only 18% (30 out of 165) of patients had a FRAX score recorded, and approximately 35% (57 out of 165) had their vitamin D levels checked, with 60% (99 out of 165) of those not receiving any replacement therapy. Furthermore, 64% (105 out of 165) of patients did not receive appropriate bisphosphonates. Some improvement was noted in the prescribing of bone protection for NOF fractures; however, approximately 80% of patients (104 of 131, as 34 patient were started whilst inpatient) were still not started on bisphosphonates by their GPs after discharge, and the completion rate for DEXA requests and metabolic bone clinic referrals remained low (Figure 2).

In the second cycle, out of 165 patients, 30 had gastrointestinal conditions, while hypertension and diabetes appeared in 35 and 45 patients, respectively. The prevalence of dementia increased to 20 patients, and 18 patients were under treatment for COPD. Rheumatoid conditions were seen in 22 patients, and 10 patients were undergoing corticosteroid therapy. Among the cohort, 18 patients were smokers, contributing to elevated fracture risk. Medication analysis showed that 50 patients were taking proton pump inhibitors, while aromatase inhibitors were seen in only five patients. HRT remained unused during this cycle; however, androgen blockers were being taken by three patients. These findings show the challenges faced while managing fragility fractures due to multimorbidity in elderly patients. It reinstates the need for robust local hospital guidelines and additional clinics for specialist interventions.

There were some slight improvements in the second cycle. A significant issue identified was that many patients discharged from the Emergency Department (ED) were not assessed for fragility fractures and left without proper referrals. To solve this, it was suggested that the flowcharts and protocols created during the first cycle be formalized into a Standard Operating Procedure (SOP) for the trust, guaranteeing simple access for all departments. Furthermore, it was recommended that electronic alerts can be used to notify when antiresorptive medication initiation is pending, prompting them to order DEXA scans and begin therapy.

Another contributing factor was the osteoporosis clinic's delayed opening, which limited its influence on referrals for fragility fractures by taking about six months after the first cycle. To ensure that the clinic could effectively address referral bottlenecks, efforts were made to allow it to function at full capacity. With all these interventions in place, further reaudits will be done to evaluate the outcomes as well.

## Discussion

In the UK, the elderly population is growing significantly. Just 11% of the population, or about five million people, were 65 years of age or older in 1948, when the NHS (National Health Service) was founded [17]. This number has essentially doubled over the decades, reaching 18% and over 10 million by 2023-2024 [18]. Osteoporosis is the most common bone disease, and with the aging population, its incidence is rising rapidly [11]. Despite being treatable, osteoporosis often goes undiagnosed, leading to under-treatment and increasing awareness among doctors and patients can help curb this problem [15]. While secondary prevention with bone protection therapies is proven beneficial, a small percentage of patients currently access these interventions. Implementation of established guidelines, such as those from the National Osteoporosis Guideline Group (NOGG) and NICE, remains inconsistent [11,16].

Management of fragility fractures and osteoporosis largely falls within the jurisdiction of general practitioners and orthopaedic fracture clinics. Our study indicated limited requests for DEXA scans and referrals to metabolic bone clinics. Long wait periods for metabolic bone clinic follow-ups, limitations in fracture clinic capacity and contemporary strains on general practitioners can all be blamed for this predicament, which frequently leads to missed or postponed treatment chances.

The use of bisphosphonates, like alendronic acid, following an acute fracture raises concerns because they may inhibit bone turnover, which could potentially delay healing [19,20]. However, a 2019 randomized controlled trial supported the use of bisphosphonate medication in the management of osteoporosis even in acute situations by finding that it did not delay fracture union or affect clinical outcomes [21]. According to NOGG and NICE guidelines, the first line of secondary prophylaxis for fragility fractures should be the initiation of bisphosphonate medication [11,16]. However, there are drawbacks, restrictions and possible drug interactions associated with oral bisphosphonates, which are especially problematic for the elderly. Although they are uncommon, side effects such as atypical fractures and jaw osteonecrosis are serious and should be discussed with patients in advance [22].

Fragility fractures can happen without any obvious damage or after low-impact falls. Making educated decisions about vitamin D supplementation and bone protection requires proper use of the FRAX tool, evaluation of vitamin D levels and radiological examinations such as DEXA scans. According to data from our audit, several patients who had previously been prescribed alendronic acid decided to stop taking medication because of gastrointestinal adverse effects. These patients were waiting to be referred to metabolic bone clinics, which frequently have lengthy waiting lists. Since the first two doses of denosumab must be given in a hospital, patients with chronic kidney disease (CKD) who were unable to start bisphosphonate therapy were not given alternative treatment like denosumab in primary care because of prescription restrictions. Furthermore, hospitalized patients who get their initial IV zoledronic acid dose must be monitored for follow-up doses within a year. Notably, three patients in the second audit cycle refused therapy because they were worried about the uncommon side symptoms linked to jaw osteonecrosis [22].

This audit has certain limitations, including its retrospective nature and reliance on electronic records, which may omit some critical clinical information. Also, as the study is based on a single centre, the generalisability of its findings can be debated.

**Figure 3:**
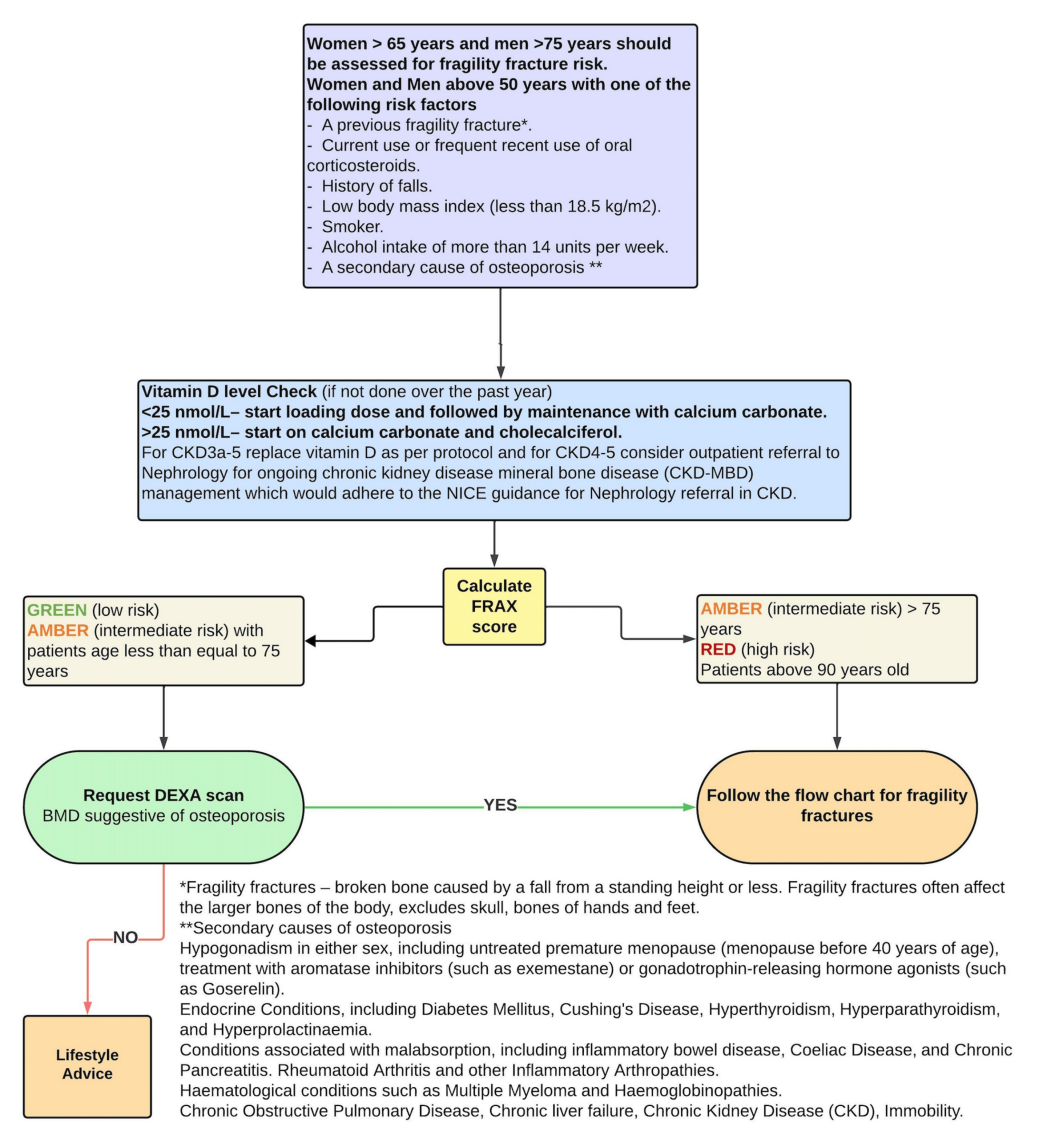
Local protocol flowchart for the management of fragility fractures (Chart 1) This local protocol was suggested after first audit cycle and further revised after second cycle. This was framed to help healthcare practitioners better manage inpatients with osteoporotic fractures. CKD: chronic kidney disease (stages 1-5); FRAX: Fracture Risk Assessment Tool; DEXA: dual-energy X-ray absorptiometry; BMD: bone mineral density.

**Figure 4:**
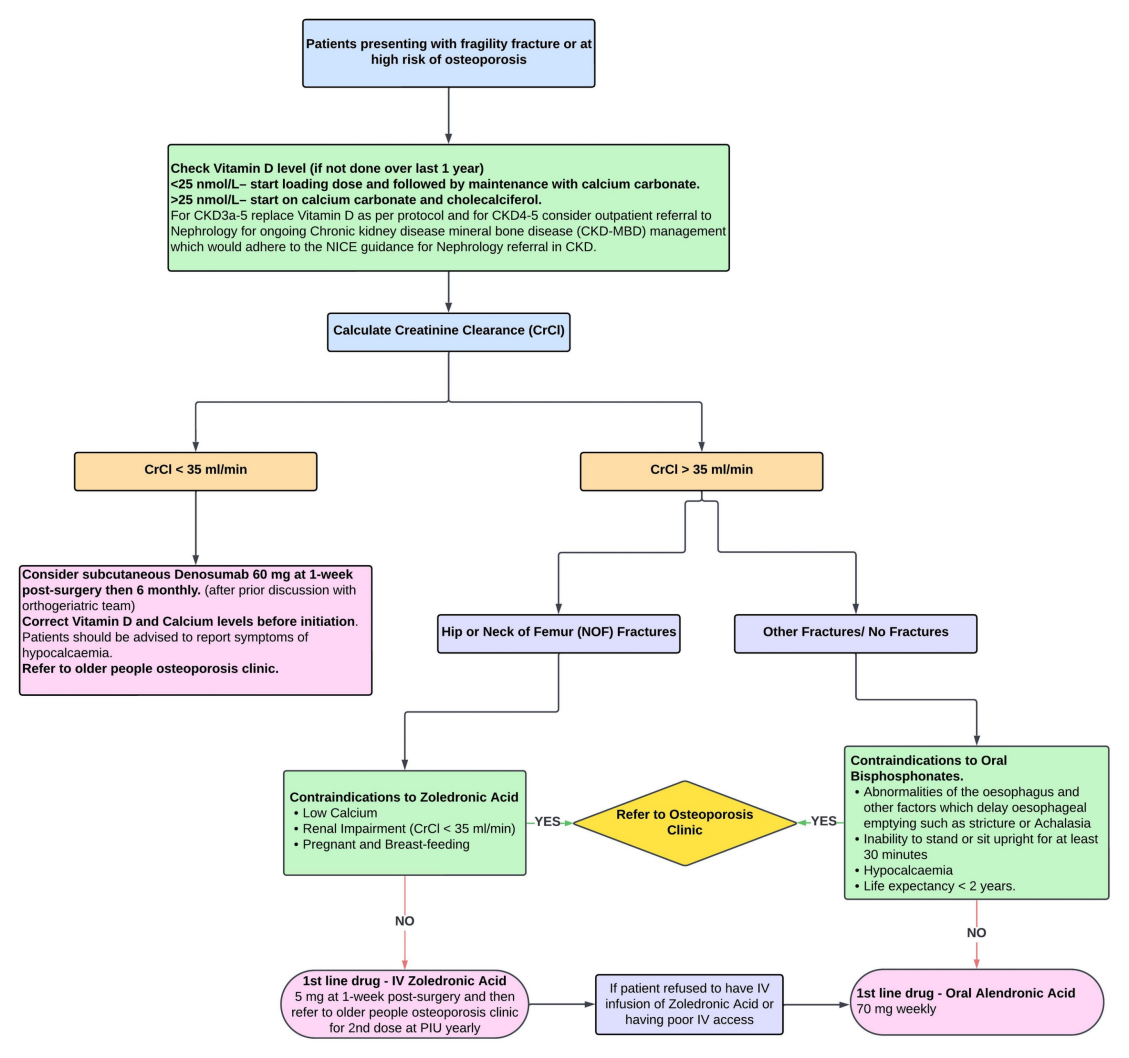
Local protocol flowchart for management of fragility fractures (Chart 2) CKD: chronic kidney disease (stages 1-5); IV: intravenous; PIU: planned investigations unit; NICE: National Institute of Health and Care Excellence.

After the first audit cycle, we suggested setting up an osteoporosis clinic for an increasingly frail population who are more likely to experience polypharmacy. This program simplifies the treatment of osteoporosis and reduces the strain on metabolic clinics. To educate resident physicians on the services that are accessible and the proper referral procedures, protocols to standardise care were developed in our trust after multidisciplinary team meeting (Figures 3, 4). Care standards can be further raised by improved coordination and communication with orthopaedic surgeons in the management of fragility fractures. Additionally, in order to familiarise emergency department staff, acute medical unit staff, orthopaedic teams and allied healthcare practitioners with osteoporosis management guidelines and relevant interventions, it is imperative that they receive targeted education.

## Conclusions

This audit highlights various shortcomings in the UK healthcare system when dealing with the management of fragility fractures. In both cycles of the audits, there was an indication of poor treatment rates in the hospital in spite of previous studies showing a high risk of further fractures and explicit guidelines supporting the early initiation of antiresorptive therapy. Reducing the morbidity and healthcare burden related to fragility fractures requires addressing these systemic concerns through greater patient education, standardized care pathways and increased interprofessional engagement.
